# Contributions to the History, Diagnosis, and Management of Croup

**Published:** 1853-02

**Authors:** John Ware

**Affiliations:** Professor of the Theory and Practice of Medicine in Harvard University


ART. II.—*Contributions to the History, Diagnosis, and Management of Croup.* By JOHN WARE, M. D., Professor of the Theory and Practice of Medicine in Harvard University.

(CONCLUDED.)

The question now presents itself, what are the grounds for believing that the two forms of the disease which I have distinguished as membranous and inflammatory, are not the same in different degrees or in different stages? and may not pass one into the other? The grounds are—

1. The very great preponderance of fatal results in the membranous croup, and a similar preponderance of recoveries in the inflammatory, and the evidence which exists that in the few cases of recovery from the former, the membrane has been formed, and in the few cases on record of death from the latter, that a membrane has not been formed—afford strong reason for believing that the diseases are essentially different. *

---

*No fatal cases having occurred of inflammatory croup under my own notice, I am happy to be able to avail myself, in support of the views above taken, of an account of four such cases, contained in the first volume of the New England Journal of Medicine and Surgery, by James Jackson, M. D., formerly Professor of Theory and Practice of Physic in Harvard University. The symptoms in all these cases were unquestionably those of croup. In one of them bronchotomy was performed.

In the first case, "the mucous membrane of the larynx was much inflamed, and smeared over with a quantity of loose mucus, but without any false membrane. The inflammation extended into the trachea as far as could be examined without opening the chest."

In the second case, "the appearances of the larynx were the same. The lungs were fuller of blood than usual."

In the third case, "there was not any coagulable lymph, the mucous membrane was highly inflamed and swollen, and the rima glotidis was very much narrowed. The membrane was smeared over with a thick mucus."

The fourth case I give at length in the words of the author:

"I was called to this on Sunday, July 5, 1812, at 3 o'clock, P. M. The disease had commenced 20 hours before, and was very strongly marked. The symptoms were considerably mitigated after vomiting. I tried in vain to take blood; the child was very fat, and the veins were all hidden, even the external jugular. The respiration grew bad again before morning, but the patient lived till the next morning, the 7th, so that the disease continued two days and a half, or sixty hours. 18 hours after death, Dr. Bigelow examined the body, and the following is his report of the appearances: "The trachea with the larynx was removed. The whole tube was pervious as usual, excepting the presence of a large quantity of mucus of the ordinary consistence. On dividing the larynx and trachea at the posterior side, and exposing the internal surface, the mucus being removed, a number of distinct red spots were discovered, of considerable size, on the lining membrane. One of these was immediately below the glottis.

2. The formation of a false membrane does not seem to require either an advanced stage or a very intense degree of the inflammation from which it proceeds. It is rather the result of a peculiarity in the kind of inflammation, than of any period or degree of it. It appears to be a very early product of the inflammation, if it be not indeed almost contemporaneous with it. It resembles in this respect the similar effusion taking place on the serous membranes, which in them occurs very early, and has ever been supposed to be the first act of inflammation. In the common inflammation of the tonsils which is accompanied by this symptom, a layer of lymph is observed to be effused over the surface of the part as soon as any signs of disease exist.

3. The circumstances attending recovery from simple inflammatory croup differ materially from those which accompany recovery from membranous croup. In the former the amendment is rapid and speedily completed. There is left behind only a moderate soreness of the larnyx, and, in the worst case, some hoarseness. There is at no time any copious or solid expectoration. In the latter, recovery is slow, unequal, and accompanied by phenomena which must necessarily attend the separation of the membrane, and the process through which the diseased mucous surface must go in order to its restoration to a healthy condition. The natural cure of the disease takes place by the occurrence of the suppurative inflam-

---

Between the mucus and the lining membrane there was no factitious substance whatevor, nor any appearance the least resembling the membranes which I have seen formed in some other cases of croup. The lungs were not examined.'

"In the other cases I had thought it possible that the disease had not continued long enough to allow the effusion to take place, as the patients all died in less than 48 hours from the attack. But in the last case such a supposition cannot be admitted ; for I have in my possession a preparation in which the false membrane is exhibited in great perfection, and this came from a patient of Dr. Channing which I had seen with him, and in which death had occurred in about 30 hours after the seizure."

The history of these cases, especially with the authority upon which they are recorded, affords very satisfactory evidence of the existence of a class of cases like those which have been above described, of a disease with the symptoms of croup, but without the formation of a false membrane either in the air passages or upon the visible parts of the throat.

mation upon the diseased surface, by which the false membrane is thrown off, and the mucous membrane then gradually returns to its natural state. In examinations after death, we usually find that this process has begun in the trachea, the membrane being there separated and often broken up into shreds, whilst the inflamed surface is covered by a layer of pus. Above in the upper part of the larynx, around the glottis, the false membrane usually remains closely adherent. It is obvious that recovery might always take place, could the parts be spared long enough from their functions to go through the necessary steps—and it is also obvious when it does take place, that it must be accompanied by a copious expectoration of pus, and of the membrane either in pieces, if firm enough, or else broken up and partially dissolved by the pus. Now these appearances do not accompany recovery from even the severest cases of the inflammatory croup, whilst they do accompany recovery from well-marked cases of the membranous form.

Of the three cases of membranous croup which are noted as having recovered, there are but two of which I have such an account as would justify me in presenting them as fair examples of the processes through which the parts pass in recovery. These were both of the most decided character and had arrived at that stage of the disease in which we expect a fatal event to occur almost from hour to hour. In the first of them, six days elapsed before any sensible mitigation of the symptoms, and even then the progress to recovery was very slow and apparently doubtful. Improvement was attended by a copious muco-purulent expectoration, in which it is true no large pieces of membrane were ever detected, but of such a consistence and appearance as would favor the belief that the membrane had escaped in a comminuted or partially dissolved state. After the probable removal of the membrane, there was for some days a bloody expectoration, the voice did not return, and it was indeed many weeks before it resumed its natural tone.

In the second case, a considerable portion of the membrane was spit up in a tubular form, after a violent fit of suffocative cough, and this was followed by the rejection of smaller pieces, mixed with a muco-purulent, at first, and then a bloody expectoration. There continned an entire loss of voice for more than a week, and for at least ten weeks after recovery it had not regained its natural tones.

The contrast is very striking between the protracted character of these recoveries, and the speedy return to health of all those who labored only under the other forms of the disease, however severe.

The observations to which the preceding remarks relate, were all made in this city and its immediate neighborhood; how far they correspond to the disease as it appears in other places, must be left to others to judge. So far as they go, they appear to me to justify the following conclusions:

1. That the only form of croup attended with any considerable danger to life, is that which is distinguished by the presence of a false membrane in the air passages.

2. That the existence of this membrane in the air passages is in a very large proportion of instances, indicated by the existence of a similar membrane in the throat.

3. That this affection differs not in stage or degree, but in kind, from all the others cases which are commonly known by the same name, and that the latter have no tendency to become converted into or to terminate in the former.

As my intention has not been to write a complete history of croup, I have omitted all such notices of the symptoms, cause, morbid anatomy, &c., of the disease as have no direct bearing on that point in its character which it was my desire to illustrate. It may not be amiss, however, to record, in connection with this paper, a few circumstances with regard to its history, which have been incidentally determined from an examination of the cases before us.

Croup is often regarded as a disease which attacks suddenly and violently. This is only true of the milder forms. Genuine or membranous croup is commonly rather gradual in its approach, and consequently often insidious. It supervenes often on the common sore throat of children; and in such cases, though its development is frequently rapid and apparently sudden, yet a careful examination of the past history of such a case, will generally satify us, that although it may have had a sudden outbreak of violence at the time it was supposed to begin, yet that it had really been coming on for several days. Of 30 cases in which I have had an opportunity of determining the mode of attack, in only two could it in any proper case be called sudden, although in many, the attention of friends was called to it quite unexpectedly, by a rapid increase in the violence of the symptoms. A sudden and violent attack is, therefore to be regarded as affording a favorable indication of the character of the case in which it occurs. The unexpected manner in which croup sometimes steals upon the common sore throat of children, should lead always to the careful inspection and watching of such cases. It is true that but a very small proportion of them do terminate in this way; but as it is the only considerable source of danger, and the only way in which they are likely to have a fatal termination, the possibility of such a course of things should not be overlooked. No case of this kind can be regarded as entirely safe from such a result. The danger is even not confined to childhood. Two of the above-named cases of fatal croup, occurred in females of 12 years of age, in which it had supervened on this affection of the throat.

The membranous croup also sometimes occurs as a sequel to the affection of the throat in scarlatina. The most common primary affection of the throat in this disease, is of the same kind with that denominated the ulcerated soar throat, viz., an inflammation, with an effusion of false membrane upon the parts inflamed. When croup supervenes upon this, the case is usually very rapid and invariably fatal. Of the cases above enumerated, two were of this character. A third occurred to me, not enumerated among them, in which there was no symptom of croup during life, the patient apparently dying from affection of the brain, but in which the usual appearances of croup were found after death. The subject of this was a young man 17 years of age. These cases all occurred between eight and ten years since. None have been observed during the more recent periods of the prevalence of scarlatina.

Croup varies considerable in its duration ; I mean its duration after its characteristic symptoms are fairly developed and there is reason to believe that the membrane is formed. Of 23 cases,

| | | |
|---|---|---|
| 1 | continued | 1 day from distinct croupy symptoms |
| 6 | " | 2 to $2\frac{1}{2}$ |
| 9 | " | 3 to $3\frac{1}{2}$ |
| 3 | " | 4 |
| 1 | " | 5 |
| 1 | " | 9 |
| 1 | " | 11 |
| 1 | " | 19 |

Nineteen cases, or more than three-fourths, therefore, were of four days duration, or less. * * * *

II.—*Treatment of Croup.*—Read before the Boston Society for Medical Improvement, by JOHN WARE, M. D., Nov. 11, 1844.

The history of the case of croup reported at the last meeting,* which I had an opportunity of witnessing during

*This was the case of a child with membranous croup, communicated by my brother, Dr. Charles Ware, of this city, in which the anodyne its progress, has confirmed me in an opinion I have, for some time, been disposed to entertain, that the methods of treating this disease in common use, require a careful re-consideration. This opinion is connected with, or perhaps has proceeded from, certain views concerning the distinctive character of various forms of disease which ordinarily are included under this one common appellation, and which I have formerly communicated to the Society. It is not too much to say, that the received mode of treating these cases, which, so far as I know, is very much the same for all their varieties, has come down to us by a sort of tradition from our predecessors. It is true that in single cases and by particular individuals, there have been occasional variations from the established practice; still in the main, emetics and bleeding, blisters and calomel, have been the principal remedies. The depleting, reducing and perturbating methods is that on which dependence has been chiefly placed.

That this treatment may be applicable to a very considerable proportion of the cases which pass under the common denomination of croup, I am not prepared to deny. Those which in a preceding communication have been classed as inflammatory, spasmodic and catarrhal, certainly recover under its influence, and apparently with greater speed than if left entirely to the resources of nature. So far as my experience has gone, however, it has appeared to produce no impression upon those in which there is satisfactory evidence that a membrane has been formed.

These cases, I should repeat the opinion expressed in the paper just referred to, are essentially of a distinct nature from the others, and constitute but a small propor-

---

treatment was mainly employed, and in which the membrane was separated and thrown off. Everything promised favorably for recovery so far as croup was concerned, but the patient died ultimately by the rapid supervention of inflammation of the lungs.

tion of those which are usually regarded as croup. They are not aggravated cases of the same kind as the others—cases which have gone to an ulterior stage of disease—but in their origin and conception different. The inflammation which is essential to them is peculiar in its character; the effusion of false membrane is not the result of an advanced stage of it, but it is one of its early results—is perhaps the first visible act of its existence; as there is much reason to believe that it is of serous membranes. It has been common to describe the stage of effusion in croup, as preceded by one of longer or shorter duration—a formative stage. If I am right in the views taken of the character of the disease, this distinction is made by making up its history from different sets of cases—going to one for the history of the first stage, and to another for the history of the second. The same confusion of diagnosis has given also an apparent success to means used for treatment. Where all the different cases which have been referred to, are grouped together as examples of the same disease in different stages or degrees, the proportion of recoveries will not appear discouragingly small. If we were to class together, as cases of consumption, all those in which there was cough and expectoration, as is done by those who profess to cure this malady, we should have no reason to be disheartened with regard to its curability; and, in the same way, so long as we class all cases together as croup, which have a croupy cough and some difficulty of breathing, the amount of mortality will not be greater than in other acute diseases of children. A more accurate diagnosis will, I am convinced, put an end to our complacency on this point. Membranous croup unquestionably does sometimes come to a favorable termination; but recovery is comparatively so rare, it forms so much the exception, that, admitting the distinctive character of the disease, it is difficult to conceive that the treatment has anything to do with the recovery. Where, under any given method of treatment, but one case out of six or eight recovers, one must be very sanguine indeed to attribute much influence upon the result to the remedies.

The question then properly arises—if the mode of treating croup commonly adopted does no good, are we sure that it does no hurt? This is a question we are far too unwilling to put to ourselves. What will happen if nothing be done? This should always be the first thought of the physician, in each individual case. Till he knows this, he cannot know with certainty what effect his treatment has; and just in proportion to the amount of his knowledge of the natural history of disease, and of the time and mode of its natural termination in recovery or death, will be his power of judging of the influence of treatment upon the result.

Now when we examine the cases of recovery of membranous croup which actually take place, and compare them with the condition of the parts in those which are examined after death, we find very fair evidences of a tendency in the disease to go through a certain course of changes which will terminate in health. The false membrane is effused, and, at the same time, the mucous membrane is thickened and congested. After a time, a process of suppuration is established upon the surface of the mucous membrane, underneath the false membrane, which of course separates the latter from the former, so that it lies loosely upon it, whilst between them is a layer of pus. If the membrane thus thrown off be thick and strong, it is expectorated in distinct pieces, sometimes of a considerable size; if it be thin and less firm, it is either converted partially into pus, or else is broken up into smaller shreds and mixed with the pus, so as not to be distinguished from it, except by very careful examination, and thus it is gradually thrown up. The diseased membrane does not free itself from the false membrane over its whole surface at once. Those portions from which the false membrane has separated, are left in an inflamed and irritable state—the expectorated membrane and pus are often tinged with blood, probably from the fact that by the violent effort of coughing some portions are torn off from the mucous surface before the purulent process had effected a complete separation. The cough, then, with more or less expectoration, and a hoarseness, in some cases amounting to an incapacity for speaking except in a whisper, continue for some time—the affection of the voice for several weeks. The parts are at length, however, perfectly restored.

In cases which prove fatal, we find evidences that the same succession of changes is taking place; that an effort has been making to bring about the same result. It is in fact from the examination of the progress which has been made in fatal cases, that we are enabled to judge what is the exact condition of the parts, and what the processes through which they go, in those which recover. Thus in some portions of the organ affected, we find the false mem brane very closely adhering to the mucous, whilst the latter is reddened and thickened. This especially occurs at the top of the larnyx. Lower down the false membrane is more or less extensively loosened from its adhesion—usually irregularly so—whilst a layer of pus lies between it and the mucous membrane. In some places the effused coat has been entirely separated, and has been either spit up, or else is found loose, enveloped in pus, in some part of the passage; whilst the surface to which it adhered is red, swollen and besmeared with pus. Thus we trace everywhere distinctly the existence of a process the tendency of which is obviously to bring about recovery; but death has taken place before it has been completed. It takes place in different steps of the process. Sometimes quite early, before any separation has taken place, the patient apparently dying from the diminished aperure of the air passages from spasm and inflammation.

Sometimes later, when the separation has taken place below, but not at the top of the larynx. At other times the membrane separates in considerable quantities, becomes collected into considerable masses, and produces suffocation by being wedged in at the bifurcation of the trachea or at the very top of the larynx. There are other cases in which recovery is also obviously taking place from croup, but iu which death occurs from the supervening of secondary disease in the lungs.

Croup, when once established, can then only be recovered from, by going through with this regular course of changes. These are essential to it. When once this process has begun; when the false membrane has been fairly effused, the parts can no more recover without them than the eruption of small pox can be cut short in its progress. A rational method of treatment, then, is that which will promote the necessary changes. And what do we need? 1. To prolong life, to prevent suffocation, in order to give time for the required process to be completed by the efforts of the organs themselves; and 2. To use means which will promote and hasten this process—which will aid the system in the work which she is aiming to perform.

Now are the usual means likely to answer these purposes? Have they answered these purposes? That emetics and bleeding sometimes relieve violent turns of dyspnœa, must be admitted; yet that they actually prevent suffocation in many cases, admits of very great doubt. But do they contribute at all to those changes upon which alone we can depend for actual recovery! There is no evidence that they do; whilst on the contrary there is reason to fear that they may interfere with them, may retard them, may prevent them. If, then, these remedies be at best of doubtful efficacy, is it not right, in so formidable a disease, to make the trial whether other measures may not be more successful? At any rate, if other means are not more successful, they may at least be less tormenting to the patient, and inflict a less amount of unnecessary suffering.

It is to be remarked of the case which has suggested these observations, that the subject of it rejected all remedies, so that it was in fact a case left very much to the resources of nature. Still, so far as the morbid condition in which croup consists is concerned, recovery was very fairly taking place, and would have been complete, except for the occurrence of a secondary affection. I may say also of the very few cases which I have seen completely recover by the expectoration of the membrane, that they were not the subjects of very active perturbating treatment, especially after the first stages had gone by, but were left a good deal to palliatives—to mild, soothing applications. It would seem worth while therefore, to make the attempt of treating the disease without the persevering use of the heroic remedies by which it has been ordinarily encountered; that we should—not perhaps leave the disease wholly to nature—but trust it at least to such remedies as will not interfere with that regular course by means of which nature is always attempting to give relief.

III —*Further Remarks on the Treatment of Croup.* Read before the Boston Society for Medical Improvement, Feb. 20, 1845.

Some remarks were presented to the Society, a few months since, on the treatment of croup, including suggestions concerning the management of that form of the disease which is attended by the formation of a false membrane in the larynx and trachea. A case of the disease has since occurred to me, which seems to be worthy of notice in connection with those remarks.

The subject was a male 5½ years of age; of pale and delicate aspect, and slender habit. He had not been perfectly well since an attack of scarlatina, two years ago; since then he had been frequently liable to colds, with severe coughs. He had enlargement of the submaxillary glands of the tonsils.

He was first seen on Sunday eve, Feb. 9, 1845. The account given by his parents was, that he had had a cough with a croupy sound—a sound with which they were familiar—for ten days past; but with it no trouble in breathing; that to-day, however, his voice had become hoarse, and that he had several turns of hard, suffocative breathing. The cough and respiration were at this time distinctly those of croup, though at the time of the visit there was no distress. There was false membrane on the tonsils. He had taken an emetic of ipecacuanha and a dose of castor oil.

He was directed to take, once in three hours, 1½ grains of Dover's powder and half a grain of calomel—to sponge the neck frequently with warm water, and to apply to it this liniment—℞. Olei oliv., ℥j.; aquæ potass., ʒij.; ung. hyd. fort., ʒj. M.

Feb. 10th.—The night had been easy upon the whole, though there had been several turns of distress. During one of these he took two drachms of wine of ipecac., with free vomiting. The symptoms of the membranous croup were perfectly well marked, but there was no distress. The liniment was continued, a flaxseed poultice was applied to the neck, and the powders continued every two hours; to be suspended, however, if he became fully opiated.

During the day the voice became quite extinct; and the cough lost the loud and ringing sound which it presents in the early period of this disease. The breathing became more labored, and was accompanied by greater muscular effort both in inspiration and expiration. Still he was not distressed, owing apparently to the influence of the opium. The air, entered the lungs well. There was much sound of loose secretions in the larynx and trachea, but no expectoration, except of a little frothy mucus. It having been found difficult to keep the poultices in contact, the parents substituted boiled mullen leaves, which were assiduously applied. At the same time the patient was made constantly to inhale the vapor from a boiling decoction of the same plant, and this was persevered in uninterruptedly for several days.

It is not necessary to follow up a detailed history of the case. These measures were continued without change for several days, i. e., the poultice, the liniment, the inhalation, and the calomel and opium in sufficient quantities, to keep him under a moderate narcotism.

On Feb. 12, Wednesday, there had been no distress of breathing; but its croupy character still continued; there had been no return of natural voice; but the sound of the cough had changed, and was like that of common catarrh—quite loose. Through Wednesday and Thursday, there was much rattling of loose matter in the larynx and trachea, and it was coughed up in considerable quantities. Portions of the sputa were mixed with blood, and false membrane was detected in detached pieces enveloped in mucus and pus. One portion of it was of considerable size and distinctly tubular. The fits of coughing, especially when masses of false membrane were ejected, were suffocative, and the sputa were dislodged with difficulty. On Thursday, there was still a large thick patch of false membrane on the tonsils. He was occasionally delirious. The pulse was about 120; the respiration varied from 12 to 20, and continued distinctly croupy, though without any distress. He was extremely prostrated.

On Saturday the respiration had lost the croupy character, but there was still a loose rattling sound in the air-passages, and the voice was unchanged. This day, for the first time, he manifested a little appetite, and his tongue became clean. He had continued occasionally to throw up pieces of false membrane.

On Monday, Feb. 17, he appeared perfectly well except as to strength and voice. By considerable exertion he could make a slight approach to proper voice, but for the most part he spoke in a whisper. *

The important point to determine in connection with this case, is, how far recovery depended upon the treatment. The treatment consisted—

1. In the absence of all reducing, depleting, and disturbing remedies.

2. Keeping the patient under the full influence of opium combined with calomel.

3. Constant external application of warmth and moisture, and of a mercurial liniment slightly stimulating.

4. Constant inhalation of watery vapor.

It is too much to say that the recovery in this case was to be attributed, with anything like certainty, to the mode of treatment employed. It may have been only one of those coincidences which so frequently mislead us in studying the effects of remedies. Still, as the expectoration of the false membrane has not been a very common occurrence under my observation, and recovery not universal even where it has taken place, it will be at least useful to notice the circumstances which have accompanied a favorable case.

On the suppposition that the successful result may have been connected in some degree with the treatment, I should be disposed to attribute it to the following circumstances:

1. To the absence of all such measures as tend to irritate the parts inflamed, and thus to interfere with the natural process of restoration—especially vomiting. That vomiting gives relief to the paroxysms of bad breathing in croup, will not be doubted; and so does it give tempo-

---

* This patient has had no return of the disease to the presen. time, March, 1850. His voice was not perfectly restored for many weeks.

rary relief to the distress of an inflamed stomach. But relief of a symptom is not the cure of disease, and does not always tend to its cure. It is not in accordance with what we know of the effects of remedies in other inflamed parts, that concussion, motion &c., should allay their inflamed condition. Vomiting relieves inflammation of some parts, and some kinds of inflammation; but in this case the parts inflamed are mechanically disturbed by the act, and it has so far as we can judge, no probable influence upon that peculiar condition which constitutes the disease.

2. To the absence of all depressing and debilitating remedies—as bleeding, purging and vomiting, considered in their effects upon the system. Such means may be beneficial when we expect resolution of an inflammation. But where the successful issue of the disease depends upon its going through with a certain course of changes, as in croup, they are as likely to interfere with as to promote them.

3. To the relief of the spasmodic contraction of the rima glottidis, which seems more or less to accompany its mechanical diminution by the effused membrane, and to aggravate very much the difficulty of breathing. It is probably upon the suspension of this spasmodic condition that the temporary relief produced by vomiting chiefly depends, and especially vomiting by means of tobacco.

4. To the influence of external warmth and moisture in promoting the suppurative process, by which alone the false membrane can be safely separated.

5. To the constant inhalation of watery vapor. This may have promoted the separation of the false membrane by keeping it from becoming dried by the constant passage of air—and by rendering it pliable and soft, so as to be easily managed and expelled by the organs in the act of coughing.

These considerations lead to the belief that this method of treating croup is at least worthy of trial. But even should it not prove more successful, it is certainly vastly more comfortable than the ordinary method. The patient, whose case has been recorded, suffered very little after the first day, even before the extrication of the membrane. Inded, taking the disease altogether, it was not attended by more distress than accompanies the average of the acute affections of children.

IV.—*Additional Remarks on the Treatment of Croup.* Read before the Suffolk District Medical Society, March, 1850.

Since the occurrence of the case described in the foregoing paper, I have had, from various circumstances, fewer opportunities of witnessing cases of croup than in former years, and only five of this form of the disease have fallen under my notice. The three first of these were treated in the method pursued in the case above related.

The first case was that of a male, 4 years old, who was taken with membranous sore throat accompanied by high constitutional irritation, Oct, 14, 1845. No croupy symptom occurred till Oct. 18, when they were manifested in a perfectly distinct manner. On the 20th and 21st, patches of false membrane with bloody sputa were raised—and one piece of four inches in length. The raising of the latter was accompanied by a severe and suffocative paroxysm of coughing. On the 22d he died, eight days from the commencement of the disease, and four from the access of croup. The suffering in this case was very considerable, but far less than I have been accustomed to witness in cases of croup treated according to the ordinary method.

The second was that of a female, 4 years of age, taken with croup on the 8th of Nov., 1845. No depleting or reducing remedies were employed. Patches of membrane, and one piece of considerable size, were brought up on the

10th and a few following days. She never suffered much, improved steadily, and on the 15th seemed well in all respects except the voice, so that on the 16th I did not see her. On the 17th there was a return of all the croupy symptoms, including the appearance of lymph upon the tonsils, and she died on the night of the 19th, eleven days after the first seizure. During no part of the disease was the suffering from dyspnœa very intense for any continued period.

On dissection, the usual appearances were found, and in one lung the false membrane extended for some distance into the bronchi in the substance of the organ.

The third case was a female, 6 years of age, who was seized with the disease Oct. 31, 1837. The onset of the disease was gradual, yet quite distinct. Nov. 2d, the symptoms had become quite severe, and Nov. 3d there was bloody expectoration and pieces of membrance were spit up. Pieces of membrane continued to be found in the sputa for several days, and she was very comfortable and breathed with tolerable ease, yet never losing the distinct croupy sound of respiration and voice. On the 8th she became rapidly worse, but without distress, and died on the 9th, quite easily, ten days from the first attack of the disease.

It will be admitted, I think, that these cases, especially the two last, exhibited certain differences from the common course of this disease, which indicated a favorable influence from difference of treatment.

In all of them the membrane was thrown up in considerable quantities.

In all of them the disease was attended by very much less distress than is usual in croup, and, in two, there was so decided a mitigation of symptoms following the separation of the membrane, as to lead to considerable hope of a favorable termination.

In two, at least, the disease was prolonged to at least twice its average duration under the usual treatment.

In the two other cases, to which reference was made, the same general course of treatment was followed, with the addition of the introduction of a sponge wet with a solution of the nitrate of silver into the larynx. In each of these cases the application was made as early in the disease as I became satisfied of its distinct character. It was repeated morning and evening. It decidedly gave relief to the breathing soon after each application, and both cases ultimately recovered perfectly. For the suggestion and adoption of this valuable addition to our means of treating this formidable disease, we are indebted, as is well known, to the enterprise of Dr. Horace Green, of New York. The profession, I think, owe to him a large debt of gratitude, for the energy and perseverance manifested in the introduction of this remedy, and I am the more disposed to render this tribute to him because so many attempts have been made to detract from his merit in relation to it.

I am well satisfied from what I have now seen of this method of treating croup, as compared with that which has been followed for so many years, that it has the advantages which were pointed out in one of the preceding papers. It is a disease which I would treat without depletion—except perhaps by a few leeches—without vomiting, without purging, without blisters, without antimonials, ipecac., and all those other nauseous remedies which have been usually resorted to. I would trust to opiates, perhaps calomel, emollients, and the local application of the nitrate of silver.

I ought to add that many of my friends in the profession have informed me of cases in their practice, treated on these principles, which have recovered in a favorable manner. Among them I would refer to Dr. Fisher, Dr. Henry G. Clark, Dr. E. H. Clark, Dr. Buckingham, and my brother, (Dr. Charles Ware,) of this city, Dr. Cotting of Roxbury, and Dr. Spooner of Dorchester.

